# Echocardiogram screening in pediatric dialysis and transplantation

**DOI:** 10.1007/s00467-022-05721-z

**Published:** 2022-09-17

**Authors:** Amelia K. Le Page, Naganandini Nagasundaram, Ari E. Horton, Lilian M. Johnstone

**Affiliations:** 1 Department of Nephrology, Monash Children’s Hospital, 246 Clayton Rd, Clayton, VIC 3168 Australia; 2 Department of Pediatrics, School of Clinical Sciences, Faculty of Medicine, Nursing and Health Sciences, Monash University, Melbourne, Australia; 3 Department of Pediatric Cardiology, Monash Heart and Monash Children’s Hospital, Monash Health, Melbourne, VIC Australia; 4 Monash Cardiovascular Research Centre, Victorian Heart Institute, Melbourne, VIC Australia

**Keywords:** Chronic kidney failure, Children, Dialysis, Transplant, Echocardiography, Cardiovascular

## Abstract

Transthoracic echocardiography is commonly used to identify structural and functional cardiac abnormalities that can be prevalent in childhood chronic kidney failure (KF). Left ventricular mass (LVM) increase is most frequently reported and may persist post-kidney transplant especially with hypertension and obesity. While systolic dysfunction is infrequently seen in childhood chronic KF, systolic strain identified by speckle tracking echocardiography has been frequently identified in dialysis and it can also persist post-transplant. Echocardiogram association with long-term outcomes has not been studied in childhood KF but there are many adult studies demonstrating associations between increased LVM, systolic dysfunction, strain, diastolic dysfunction, and cardiovascular events and mortality. There has been limited study of interventions to improve echocardiogram status. In childhood, improved blood pressure has been associated with better LVM, and conversion from hemodialysis to hemodiafiltration has been associated with better diastolic and systolic function. Whether long-term cardiac outcomes are also improved with these interventions is unclear. Echocardiography is a well-established technique, and regular use in childhood chronic KF seems justified. A case can be made to extend screening to include speckle tracking echocardiography and intradialytic studies in high-risk populations. Further longitudinal studies including these newer echocardiogram modalities, interventions, and long-term outcomes would help clarify recommendations for optimal use as a screening tool.

## Introduction

The mortality rate for children with chronic kidney failure (KF) is at least 30 times higher than their age-matched healthy peers [1]. Cardiovascular disease (CVD) is a leading cause of this excess mortality, which increases for those who survive into adulthood [2]. Even with transplantation and advances in dialysis care, CVD-associated mortality remains significant [3].

Prevention of cardiovascular (CV) events in chronic KF is a major challenge as CVD is often silent and a significant proportion of CV mortality is from sudden cardiac death [4]. A key focus of pediatric kidney research has been identification of early markers of CVD in order to understand natural history and risk factors, and to inform preventative strategies. Echocardiography has been used since the 1980s in pediatric KF research and clinical practice as a surrogate outcome measure of CV health. Its use can be described as a form of screening; however, protocols for use, and guidance on how echocardiography should inform treatment are limited.

Through this review, we provide a refresher of the natural history of CVD in chronic KF and associated structural and functional cardiac changes that can be identified with echocardiography. We then summarise established and new echocardiographic techniques, and finally we review the evidence for use of echocardiogram as a screening tool in the pediatric dialysis and transplant population. Studies of children with CKD are not included in this review.

## The natural history of CVD in pediatric kidney failure

Chronic KF has been associated with all types of structural cardiac disease including myocardial fibrosis and hypertrophy [5], arteriosclerosis [6], myocardial capillary rarefaction [5], conduction system abnormalities [4], valve calcification [7], and coronary atherosclerosis [6]. In children with chronic KF, myocardial change, typically left ventricular (LV) mass increase is most commonly seen. This is thought to be an initial adaptive response to increased cardiac workload and pressure/volume overload [8]. Progression to LV hypertrophy (LVH) however can be maladaptive with potential functional consequence especially with higher cardiac workload demands [8]. There is a complex interplay of many factors that contribute to CVD processes, and in many patients these factors have been at play from earlier stages of chronic kidney disease (CKD), further increasing longer-term risk profile [9]. A summary of CVD risk factors in chronic KF is presented below.

### Traditional risk factors

Hypertension is the most common CV risk factor present in children with chronic KF, with uncontrolled hypertension evident in 51% of children after 1 year of dialysis [10] and in 25% 5 years post-transplant [11]. Hypertension causes endothelial injury and arterial stiffness leading to an increase in peripheral vascular resistance and pressure load on the left ventricle [6]. Over time, this causes myocardial injury, ventricular hypertrophy, and remodelling. Hypertension is also a pro-atherogenic state [6].

Dyslipidaemia, another pro-atherogenic state, has been reported in 45% of the pediatric transplant population [12]. An increase in atherogenic lipoproteins is seen, with inhibition of antioxidant pathways. Pro-inflammatory effects contribute to endothelial dysfunction and vascular stiffness [13]. Disturbances in body mass index (BMI) are common in KF, with coronary artery calcification more prevalent with obesity and malnutrition [14, 15]. Metabolic syndrome is increasingly prevalent post-transplantation and this can contribute to vascular inflammation, arterial stiffness, and atheroma [16].

### Kidney failure, uremic, and inflammatory risk factors

Chronic KF is a complex state of persistent low-grade inflammation [17]. Pro inflammatory cytokines are elevated, with C-reactive protein (CRP) associated with coronary calcification in children [18], and interleukin-6 (IL-6) and CRP associated with CV mortality in adults [19]. Dialysis is an independent exacerbator of inflammation by mechanisms including peritoneal dialysate and hemodialysis membrane bio-incompatibility, and catheter-associated infections [17]. Fluid overload has also recently been associated with markers of inflammation [20].

Elevated serum calcium, phosphorus, and parathyroid hormone (PTH) are all independently linked to increased risk of coronary artery calcification [18] and carotid intima media thickness (CIMT) [21] in children with chronic KF. Increased ventricular mass can be linked to cellular growth triggered by FGF23, as a result of impaired phosphate and vitamin D metabolism [22]. In addition, there are challenges with achieving an ideal balance of treatment, with overtreatment with activated vitamin D and calcium containing phosphate binders associated with CIMT [23] and coronary artery calcification [24].

Anaemia is a frequent complication of dialysis and reduced GFR and has been associated with LVH and diastolic dysfunction in several pediatric studies [21, 25, 26]. Cardiovascular complications of anaemia are believed to be due to a chronic increase in cardiac output, poor tissue perfusion, and reduced oxygen delivery to the myocardium [27].

### Dialysis and mechanical risks

Dialysis is perhaps the most significant risk factor for CVD. Pre-emptive transplantation and reduced cumulative time on dialysis have been associated with improved survival [3, 28]. A multitude of inter-related factors are at play including exaggerated risks of traditional and uremic/inflammatory factors, and of course fluid overload. Risk is potentially most significant for the hemodialysis population, with extremes of volume overload and intra-dialytic hypotension associated with mortality in adults [29]. Temporary global or regional reduction in systolic myocardial function, called myocardial stunning, is seen frequently in children on conventional hemodialysis, and can be related to intradialytic hypotension with reduced coronary perfusion and changes in volume loading [30]. Repeated myocardial stunning may cause chronic injury, contributing in some circumstances to chronic systolic dysfunction [31].

Dialysis patients are at risk of pressure and volume overloaded cardiovascular states, both of which have been associated with increasing LV mass, hypertrophy, and functional changes including impaired ventricular relaxation (diastolic dysfunction) and ventricular contraction (systolic dysfunction). There is significant interdependence of hypertension and volume overload, although control of volume state may be the most important factor in reducing LV mass [32]. The geometry of LVH may give a clue as to the predominant risk state, with volume overload and ventricular dilatation leading to a more eccentric hypertrophy, and pressure overloaded systems characterised by a concentric hypertrophy [33]. Arterial stiffness identified by increased aortic pulse wave velocity (PWV) can contribute to this pressure overload. It is often described as a marker of vessel ageing, which is seen in children with KF [34]. With a lifetime of kidney replacement therapy ahead of them, these changes are significant.

### Echocardiography techniques and limitations

Trans-thoracic echocardiography is the most widely used diagnostic modality available for structural and functional cardiac assessment. Conventional modes of echocardiography use two-dimensional (2D) assessments of structure and motion to assess myocardial wall thickness, wall mass, chamber size, and systolic function. Accurate assessment requires gating with an electrocardiogram to determine timing of systole and diastole. Integration of Doppler measures allows evaluation of pressure, velocity, valvular function, and diastolic/systolic function (including tissue Doppler imaging TDI). Newer methods such as strain imaging and three-dimensional (3D) echocardiography may provide more accurate assessments and early detection of sub-clinical systolic dysfunction [35]. Overall echocardiogram limitations include inter- and intra-observer variation, indirect derivation of some measurements, and system and resource considerations. Specific to KF, volume state dependence of some measures needs to be taken into consideration. A summary of echo modalities, clinical utility, and limitations is discussed below and further detail of commonly reported parameters in chronic KF publications is provided in Table 1.

**Table 1:** Clinical and research echocardiogram measures relevant to pediatric chronic kidney failure *associated with adult mortality or CV events

Echo pattern	Reported measurements		Details	Notes/caveats
**Left ventricular dimensions/geometry**	Left ventricular mass (LVM)* Requires measurement of interventricular septal thickness(IVSD), left ventricular posterior wall thickness (PWD), left ventricular diameter (LVEDD)		Typically 2DE or via M-mode Diameters acquired in diastole via (1) single plane, with mass calculated via Devereux formula OR via (2) two planes, using the cross-sectional formula or ellipsoid formula OR via (3) biplane Simpson method	LVEDD, IVSD, PWD absolute values can also be compared with references and presented as *Z*-scores
	Left ventricular mass index (LVMI)*		LVM can be indexed to BSA, or lean BSA, or height ^2.7^	Reference value examples see [36, 37]
	Left ventricular hypertrophy (LVH)*		Usually defined according to gender/age/BSA or height based LVMI percentile or *Z*-score	
	**Relative wall thickness (RWT)** = 2 × posterior wall thickness/internal diameter at end diastole	**Concentric remodelling**	N LVMI and RWT ≥ reference	Reference may be absolute (0.43 most commonly used) or by 95^th^ percentile
		**Concentric LVH**	LVH and RWT ≥ reference	
		**Eccentric LVH**	LVH and RWT < reference	
**Aortic dimensions**	**Aortic dilatation** Dimensions of aortic root and ascending aorta, aortic valve annulus, aortic sinus, sinotubular junction		Measurements usually reported as absolute dimensions and *Z*-score	References are age, weight, BSA, and gender dependent
**Systolic function (LV)**	Left ventricular ejection fraction* (LVEF) = (end-diastolic volume − end-systolic volume)/ end-diastolic volume × 100%		Measures the change in volume, with volumes typically acquired in 2 planes or via the biplane Simpson method	Impaired usually defined as < 51%, although references are age and gender dependent. Less interobserver differences v FS
	Fractional shortening (FS)* = (end-diastolic dimension − end-systolic dimension)/ end-diastolic dimension		Measures the change in dimensions. Diameters can be obtained by M-mode, 2D or 3D	Impaired usually defined as < 25%
	Regional wall motion* abnormalities (RWMAS)		Systolic function of individual segments of the left ventricle according to standard location and coronary distribution Can be assessed visually on 2DE or with strain imaging This is often used to assess **STUNNING** during or post-dialysis	
	LV end-systolic volume* LV end-systolic diameter*		These parameters can reveal volume loading/state, are a measure of ventricular contractility, and reflect ventricular remodelling	
	Global (systolic) longitudinal strain* (GLS)		Usually assessed by STE in multiple planes with measures calculated = end-systolic distance between two speckles of tracked endocardium minus the original distance divided by the original distance Peak values for each segment are averaged and then reported as global strain	Should be a negative value, with better contractility if values more negative Reference ranges often defined as: Normal GLS > − 18 to 20%, Reduced GLS − 14 to − 18%, Severely reduced GLS < − 10%, but use of age and vendor specific reference values is recommended
	**Global (systolic) radial strain (GRS)**			
	Global (systolic) circumferential strain (GCS)*			
	Systolic longitudinal strain rate*		Rate of LV deformation during systole Should be negative	Advantage in dialysis assessment due to relative volume independence
	**LV performance** by heart rate–corrected velocity of circumferential fiber shortening **(VCF)**		= FS in short axis/rate-corrected ejection time	
	**LV contractility**		Calculated by the difference between measured and predicted VCF for the calculated wall stress	Advantage in dialysis assessment due to relative volume independence
	**LV contractile reserve**		Difference between LV contractility at rest and peak exercise	
	mechanical dispersion index/asynchrony index* (Measure of Mechanical Dyssynchrony)		By STE Defined as the standard deviation of time to peak longitudinal systolic strain across multiple LV segments	
	**LV performance by Tei index **[38] isovolumic contraction time + isovolumic relaxation time/ejection time		Assessed by 2DE/PWD or TDI Doppler A measure of systolic and diastolic function	
**Diastolic function (LV)**	***E*/*A* ratio** (measure of relaxation)		PWD flow velocities across mitral valve at LV early (E) and late diastole (A) to assess LV filling	Normal reference usually 1–3 Can be falsely elevated in states of volume overload
	***E*/*E***′ ratio * (measure of compliance)		PWD flow velocity (E) to TDI (*E*′ lateral, septal, or averaged) ratio at the mitral valve	References dependent on age and site of TDI *E*′ measurement
	**Stiffness index** (E/E′)/left ventricular end-diastolic dimension		By M-mode and TDI	
	**Isovolumic relaxation time**		Time from end of aortic outflow to start of mitral inflow	
	LV end-diastolic volume*		A measure of preload	
	**LV diastolic strain**		Usually assessed by STE in the respective plane (can also be assessed by TDI) = end-diastolic distance between two speckles minus the original distance/original distance	Should be a positive value, with better relaxation if more positive
	Diastolic longitudinal strain rate *		Rate of LV deformation during diastole	Should be positive
	Left atrial volume index (LAV)*		Indexed to BSA	
	Left atrial strain (LAS) × E:LAS × LAS strain rate*		By STE—LA longitudinal strain. Can be measured at difference phases in cardiac cycle. The ratio of transmitral E wave: LAS assesses LV filling pressure	Should be negative, with better function if values more negative

*CV*, cardiovascular; *Echo*, echocardiogram; *2DE*, 2-dimensional echocardiogram; *LV*, left ventricular; *VM*, left ventricular mass; *LVMI*, left ventricular mass index; *LVH*, left ventricular hypertrophy; *RWT*, relative wall thickness; *BSA*, body surface area; *TDI*, tissue Doppler imaging; *STE*, speckle tracking echocardiography

### Modalities

#### 2-D echocardiography (2DE)

**Fig. 1:**
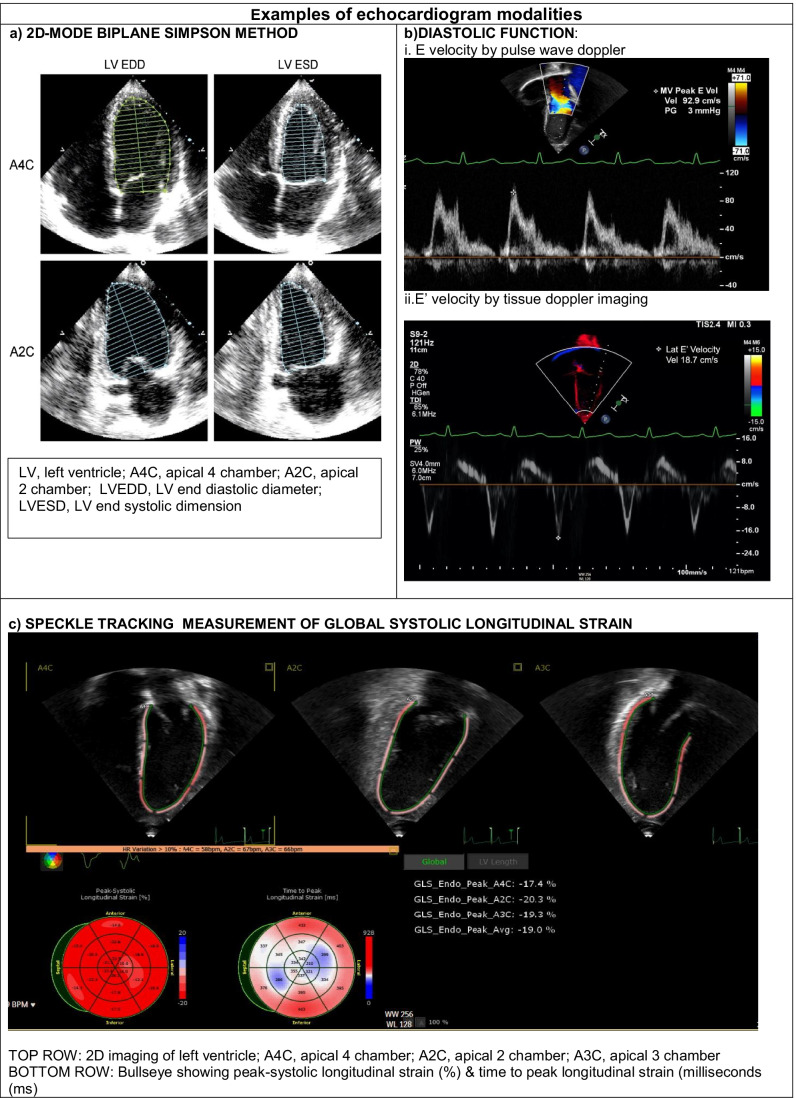
Examples of echocardiogram modalities. **a** 2D-mode biplane Simpson method. **b** Diastolic function: i, E velocity by pulse wave Doppler; ii, *E*′ velocity by tissue Doppler imaging. **c** Speckle tracking measurement of global systolic longitudinal strain

2-D echocardiography is the most commonly used modality and provides cross-sectional views for basic assessment of cardiac structure and function. Left ventricular ejection fraction (LVEF) is calculated by the biplane Simpson’s method using the formula (left ventricular end-diastolic volume − end-systolic volume)/end-diastolic volume in 2 planes (apical four chamber and apical two chamber) [39]. Systolic and diastolic ventricular volumes are measured by tracing the endocardial border of the ventricle, which is typically then divided into 20 disks (Fig. 1a). Due to the nature of the tracing, certain geometric assumptions are made, leading to potential inaccuracies in non-ellipsoid ventricles.

#### M-mode

Motion-mode or M-mode is a time gated view of structures along a single ultrasound line. It is generally used as a complementary tool in assessment of ventricular wall thickness, chamber dimension, and abnormal valvular movement. Measurements can be acquired in circumferential (short-axis) or longitudinal (long-axis) planes. Linear measurement of left ventricular end diastolic diameter (LVEDD) and left ventricular end systolic diameter (LVESD) are used to calculate fractional shortening (FS) as a measure of LV systolic function ((LVEDD − LVESD / LVEDD) × 100) [39]. However, due to the inter-observer variability and inability to detect regional wall motion abnormalities, M-mode is largely being phased out in favour of more robust and reproducible modalities such as multiplanar imaging, 2DE calculated ejection fraction, and functional analysis using strain.

#### 3-D echocardiography (3DE)

3-D echocardiography obviates the need for geometric assumptions and provides more accurate measurements of ventricular volume and function than 2DE [39]. It is, however, currently limited by quality of image acquisition and time, and is reliant on technical capabilities of the software, and thus, it is not routinely integrated into echocardiographic assessments. Automated and artificial intelligence-optimised 3DE imaging packages can improve standardisation and optimise resource utilisation but are vendor-dependent and currently used on a limited basis. Cardiac magnetic resonance imaging (MRI) also provides 3D imaging and may become standard of care; however, there are safety considerations in KF including requirement for sedation in younger children, and when gadolinium contrast is required.

#### Doppler assessments

Doppler echocardiography allows assessment of velocity and direction of blood flow to provide precise hemodynamic evaluation of the heart. Doppler velocity data is used to derive pressure data using the Bernouilli equations to estimate systemic and pulmonary pressures. Data across valves is useful to gauge valvular function and ventricular diastolic function. For LV diastolic function, velocities are measured at the level of the mitral valve, with the E wave representing early diastolic flow (Fig. 1bi), and the A wave representing late diastolic flow or the flow during atrial contraction. LV filling pressure is most often expressed as the *E*/*A* ratio, with impaired ventricular relaxation and diastolic dysfunction often defined by *E*/*A* < 1. Continuous wave, pulsed wave (PWD), and colour Doppler are the main modalities utilised. Limitations with Doppler studies include underestimation of the gradient if the sound beam is not exactly parallel to jet. In addition, fast heart rates can make assessment of A and E waves difficult. Finally, volume status can influence some parameters (typically higher *E*/*A* ratio), thus standardisation of scanning in relation to dialysis schedules is important.

#### Tissue Doppler imaging

Tissue Doppler imaging (TDI) measures myocardial velocities in specific locations. Typically, pulsed wave TDI is used. Measurements include velocity data during early diastole (*E*′), late diastole (*A*′), and systole (*S*′). Peak measurements are most commonly undertaken at the LV free wall myocardium just below the mitral valve annulus (lateral), medial wall just at the top of the ventricular septum (medial), and at the right ventricular free wall just below the tricuspid valve annulus (RV). TDI assessment of mitral annular velocity (*E*′) (Fig. 1bii) is a validated measure of LV diastolic function correlating with invasive assessment on catheter, and is less pre-load dependent than Doppler mitral inflow velocity (*E*) [40]. As with conventional Doppler, in diastolic dysfunction, impaired relaxation will decrease *E*′ more than *A*′, and TDI *E*′/*A*′ is another measure of diastolic function. Conventional *E* to tissue Doppler *E*′ (*E*/*E*′) may be the most reliable and sensitive Doppler measure of diastolic dysfunction [41]. TDI can also be used to quantify regional and global systolic LV function (*S′*), and to measure strain. There may be limitations of TDI due to discrepancies in the beam angle and limitations in plane of assessment (assessment of radial and circumferential strain is limited), although inter and intra-observer reproducibility appears reasonable [42].

#### Speckle tracking echocardiography (STE)

Speckle tracking echocardiography (STE) tracks the motion of individual reflections within the myocardium to assess myocardial deformation and provides an assessment of strain (also known as strain imaging) during systole or diastole. Strain is associated with myocardial hypertrophy and fibrosis in human and animal studies [43, 44]. Microvascular dysfunction has also been associated with impaired strain [45]. Typically used with 2DE, STE can detect myocardial displacement along longitudinal, radial, and circumferential planes. Global longitudinal strain (GLS) is measured as the relative change of LV myocardium between end-diastole and end-systole, and should be a negative value (Fig. 1c). A less negative value is indicative of worse systolic LV function. GLS is a sensitive measure of systolic function and has recently been identified in adult KF as a more precise predictor of cardiovascular mortality than ejection fraction [46]. Regional strain can be assessed by STE and has been used to help define mechanical dyssynchrony [30]. Software can also be used to calculate speckle derived ventricular volumes and derive an ejection fraction [47]. Some of the limitations with STE include its dependency on 2D image quality and frame rates, and it remains volume dependent, especially for hemodialysis patients. STE however is non-angle dependent and is mostly reproduceable. Strain measures by STE (versus TDI) are often preferred for this reason, and also because STE is less time consuming. 3D STE is an emerging new technology that may overcome some of the inaccuracies of 2D STE but is not yet validated in the pediatric cohort [48].

#### Contrast enhanced echocardiography (CEE)

CEE has been recently approved for use in children in the USA. This technique uses intravenous microbubble contrast agents in conjunction with echocardiography, and is considered to improve resolution, with utility especially in obese patients [49]. Clear resolution to trace the endocardial border may provide more accurate LV function assessment, and may be more helpful in identifying perfusion associated regional wall dysfunction [49].

#### Stress echocardiography

2DE in conjunction with exercise- or drug- (e.g. dobutamine) induced myocardial work can be used to detect coronary artery disease through detection of regional wall abnormalities. It is rarely clinically used in children but in research has been shown to demonstrate that children with chronic KF on dialysis have reduced contractile reserve during exercise and dobutamine stress [8, 50]. These authors hypothesised that this may predict future systolic dysfunction and heart failure; however, long-term follow-up outcomes have not been determined to our knowledge.

#### Novel techniques

The cardiac work index (CWI) is a non-invasive assessment of strain and LV pressure. Strain data derived from STE is superimposed on population reference LV pressure measures at time of LV peak pressure, mitral valve opening, and closure. The derived LV pressure curve is then matched with the patient’s strain data to develop a pressure-strain loop. The area of the loop is the CWI [51]. CWI can accurately and independently predict mortality in an adult hemodialysis population, and may be superior to LVEF and GLS [52]. CWI has not been assessed in a pediatric KF population to our knowledge.

### Pediatric echocardiography standards in kidney failure and reference range challenges

Wall thickness, chamber size, and functional assessment should form part of each echocardiogram assessment and there are published recommendations to guide this [39], as well as to standardise reporting [53].

Given the potential hemodynamic changes in chronic KF, echocardiogram standardisation can be a challenge [54]. Ideally, routine assessment of patients on dialysis should occur at rest, on an interdialytic day when close to or at ideal/target weight, and at target hemoglobin concentration. Ideal weight, however, can be difficult to assess and echocardiography may also be used to help define this. Inferior vena cava (IVC) parameters along with clinical correlation have been shown to accurately predict fluid status in dialysis patients with adult studies showing improved cardiac mechanics and left ventricular mass (LVM) with dry weights adjusted according to IVC diameter [55]. Additional data can be derived from echocardiograms performed during dialysis or in the immediate post-dialysis period [30, 56] and a number of methods incorporating time have been used to reduce the impact of volume status on echocardiogram measures. An example is the myocardial performance index (MPI), or Tei index. It is assessed using Doppler imaging and is defined as the sum of the isovolumic contraction and relaxation times divided by the ejection time [38]. It is considered a reliable parameter for global LV function assessment but is mainly used in a research capacity in children. Other echocardiogram confounders include age, gender, race, and body composition. To account for body composition, LVM is commonly indexed to body surface area (BSA) or height, and presented as the left ventricular mass index (LVMI). To compare with healthy reference populations, values in pediatric echocardiography have been recommended to be presented as *Z*-scores [39]. Given body composition issues in chronic KF, presentation of height or BSA-based *Z*-scores for LVM may be most accurate [57]. The most relevant reference intervals should be used which are best representative of one’s cohort, and in accordance with one’s vendor’s equipment. There may, however, still be challenges in finding the most appropriate reference for the individual given the substantial variability in the healthy population, particularly for LVM.

### Echocardiogram patterns of relevance in chronic kidney failure

An increasing array of echocardiogram changes in chronic KF are now described. In many of these, associations with cardiac events and mortality have been reported in adult populations. Longer outcome studies in pediatrics are required. Detailed definitions of more commonly described markers in clinical and research use are provided in Table 1.

### Echocardiogram studies in pediatric kidney failure

There are countless observational studies of prevalence and risk of echocardiogram changes in pediatric chronic KF. Many incorporate control populations to demonstrate significantly worse LVM, diastolic and systolic function [21, 41, 58–61]. Each publication must be interrogated closely. Demographic factors such as duration of dialysis can significantly impact findings, and definitions of abnormal values vary. Table 2 presents key studies evaluating children with chronic KF, aiming to highlight the prevalence of echocardiogram abnormalities, multivariable analysis-derived risk factors, and follow-up of echocardiogram changes over time.

**Table 2:** Echocardiogram abnormalities in pediatric chronic kidney failure – prevalence and associated risk factors

Study	Population characteristics	Echo feature/definitions/normative values	Prevalence	Significant risk factors (from multivariable regression analyses, correlation, or versus controls)
***Mitsnefes ***[62] Retrospective observational	• Maintenance dialysis *n* = 64 • HD *n* = 26, PD *n* = 38 • Mean dialysis time 1.8 ± 2.3y	• LVMI = mass/height in meters^2.7^ • LVH = LVMI > 95^th^ percentile • Severe LVH = LVMI > 51 g/m^2.7^ • LV geometry by LVH/RWT (defined by 95^th^ percentile)	• LVH 75% (85% HD v 68%PD) • Severe LVH in 41% • Abn geometry in 80%	• Severe LVH predicted by HD, as opposed to PD
***Mitsnefes ***[63] Retrospective longitudinal observational	• Maintenance dialysis *n* = 29 • HD *n* = 13, PD *n* = 16 • Mean dialysis time at 1^st^ scan 1.8 ± 1.2mo, at 2^nd^ scan 10.3 ± 2.9 mo	• LVMI = mass/height in meters^2.7^ • LVH = LVMI > 95th percentile • Significant LVMI change = > 20% from baseline echo • LV geometry by LVH/RWT(defined by 0.41)	• At baseline LVH in 69%, and abn LV geometry in 84% • No significant change in LVH, geometry, or LVMI at 2^nd^ scan	• ↑ LVMI at follow-up predicted by ↑ SBP, and initial lower LVMI • ↓ i LVMI at follow-up predicted by ↓ SBP and initial higher LVMI
***Gruppen ***[64] Cross-sectional observational	• KF commencing at age 0–14 • Transplant *n* = 110 • Dialysis *n* = 30 (19 HD, 11 PD) • Mean duration KF 18.3y (r6–30)	• LVMI = mass/m^2^ • LVH –as per Framingham study • Diastolic dysfunction by PWD • Defined as *E*/*A* ratio < 1.0	• LVH in 42.9% • Diastolic dysfunction 13% • Aortic Valve calcification 19.3%	• ↑ LVMI assoc with ↑ mean BP (mean clinic BP over 3 months) and male gender • ↓ *E*/*A* ratio assoc with older age and GFR < 25 • Aortic valve calcification associated with ↑ PD duration
***Civilibal ***[58] Cross-sectional observational	• Maintenance Dialysis *n* = 39 • Mean time on dial 4.0 ± 2.7y • 15 HD, 24 PD • Controls *n* = 15	• LVMI = mass/height in meters^2.7^ • Severe LVH = LVMI greater than 51 g/m^2.7^ (equivalent of > 99^th^ percentile)	• Severe LVH in 69%	• ↑ LVMI associated with ↑ mean indexed SBP and ↓ HB
***Bakkaloglu ***[65] Cross-sectional observational	• Maintenance peritoneal dialysis *n* = 110 • Mean time on PD 31 ± 27mo • Controls *n* = 124	• LVMI = mass/height in meters^2.7^ • LVH = LVMI > 95^th^ percentile • LV geometry by LVH/RWT(defined by 95^th^ percentile) • Diastolic function by PWD and TDI *E*/*E*′	• LVH 72.7% • 50% concentric • 22.7% eccentric • Concentric remodelling 11.8%	• ↑ LVMI associated with ↓ HB, ↑ MAP • ↑ *E*/*E*′ associated with ↓ residual urine volume
***Kim ***[59] Retrospective and cross-sectional observational	• Transplant *n* = 32 (nil pre-emptive) • Mean time since Tx was 5.1y • Mean time on dialysis 2.0y • Controls *n* = 29 Retrospective Echo on dialysis and cross-sectional echo post-transplant	• LVMI = mass/m^2^ • LV systolic function • By 2D M mode – SF (low = < 28%),EF • By TDI – LV peak GLS, *S′* • LV diastolic function • By PWD – E,A, *E*/*A*, IVRT, By TDI – *E*′, *A*′, *E*′/*A*′ • LV global function by Tei index	• On dialysis 28.6% had low FS, 7/8 normalised post KT	• LVMI ↑ dialysis and transplant v controls • LVMI corr with E, *E*/*A*, *E*′, *E*′/*A*′ • SF ↓ in dialysis v controls • Post-transplant ↑ SF v dialysis • GLS↑, *S′* ↑ post-transplant v controls • ↑A, ↑ IVRT and ↓*E*/*A* transplant v controls • ↑ Tei transplant v controls, corr with GLS
***Shamszad ***[66] Retrospective observational	• Hemodialysis *N* = 65 HD (with 287 echo studies) Median time on dialysis 1.5y (IQR 0.5–3.6)	• LVMI = mass/height in meters^2.7^ • ↑ LVMI defined by different models • Systolic function by • SF (Abn < 2 SD below age/sex) • EF (Abn at < 55%) • Diastolic dysfunction by PWD/TDI *• E*/*A* − abn < 1 or > 3, *E*/*E*′ − abn > 10	• Systolic dysfunction 24.6% • Diastolic dysfunction by *E*/*A* 26.2%, or E/e′ 16.9%	• ↑ LVMI assoc with post dialysis HTN • ↑ LVMI (model dependent) associated with ↓ lateral *E*/*E*′ and ↓ SF
***Hirth ***[67] Retrospective observational	• Transplanted in childhood *N* = 68 oAt echo (*N* = 34 < 17y, *N* = 34 > 17y) oMedian time post Tx 9.8y (r 2–28.4) oMedian GFR 57.1 + / − 18 in children and 61.5 + / − 25.6 in adults • Healthy Controls *n* = 68	• LVMI = mass/m^2^ • LVH = LVMI by age/gender • LV geometry by LVH/RWT(defined by 0.43) • Systolic function • By TDI *S′*, by STE GLS and GLS rate • Diastolic function • by IVRT, *E*/*A* ratio, E/*E*′	• LVH in children 21%, adults 18% (most eccentric)	• ↑ LVMI, RWT v controls • ↓ *S′* v controls • ↑ GLS rate associated with ↑IVRT, and ↑SBP (by clinic BP or ABPM)
***Schoenmaker ***[41] Cross-sectional observational	• KF *n* = 38 • HD *n* = 11, PD *n* = 8, transplant *n* = 19 • Healthy controls *n* = 76	• LVMI = mass/height in meters^2.7^ • Severe LVH = LVMI > 51 g/m^2.7^ • Diastolic dysfunction defined by *• E*/*A* ratio < 1.0 • septal OR mitral E/*E*′ > 95^th^age percentile	• Severe LVH 11% • Diastolic dysfunction • by *E*/*A* ratio 5% KF • by mitral E/*E*′ 29% KF, by septal E/*E*′ 42% KF	• ↓*E*/*A* in KF v controls • ↑E/*E*′ in KF v controls • DD ↑ risk associated with • older age • male gender
***Lindblad ***[60] Retrospective observational	• Transplant *n* = 44 • eGFR 54.5 [r 10–99] • 36/44 pre-emptive transplants • median time on dialysis 0.48y • median time of functioning kidney transplant of 6.5y • CKD 2–5 *n* = 34 • Controls *n* = 19	• LVMI = LVM/height^2.7^ • LVH = LVMI of > 38 g/m^2.7^ • Systolic function • By EF, TDI *S′* • Diastolic function by • E, A *E*/*A*, *E*′, A′, E′/A′, E/E′ • LV diastolic dysfunction = TDI E′ of < 2 SD or TDI A′ or PWD E/TDI E′ of > 2 SD of the reference group	LVH 26.7% in transplant Diastolic dysfunction in 23.1% (*E*′), 28% (*A*′), and 42.9% (E/*E*′) in transplant	• ↑ LVMI transplant v controls • ↑ LVMI in transplant v CKD • ↑ LVH in transplant or CKD v controls • ↑ A in CKD or transplant v controls • ↑ E/*E*′ in CKD or transplant v controls • ↑ mean BP SDS assoc with ↑ LVMI, ↓ *E*′A • ↑ BMI assoc with LVMI • ↑ E/*E*′ in CKD associated with worse GFR • albuminuria associated with ↓ *E*′
***Kaddourah ***[68] Retrospective observational	• KF *n* = 78 • HD *n* = 34 • PD *n* = 41 • Change in dialysis modality *n* = 3 • Transplant *n* = 19	• Aortic Dilation • defined as *Z*-score > 2 dimension at aortic annulus OR root at the sinus OR sino-tubular junction OR ascending aorta	• AD 30.9%	• AD ↑ risk associated with • ↓ BMI (highest risk = BMI *Z*-score ≤ –2.0) oGlomerular cause of KF • ↑ iPTH if BMI *Z*-score –2.0 to + 0.1 • AD ↓ risk associated with • BMI *Z*-score of ≥ 0.1 if DBP index < 1
***Fadel ***[69] Non-randomised interventional	• HD *n* = 30 • Duration of HD ≥ 6mo, then converted to on-line-HDF and followed for 6 mo oMean HD duration 53 ± 32 mo	• Systolic function • By EF, FS, dysfunction = FS < 28% • Diastolic function by • Mitral decceleration time, *E*/*A* • LV DD = (*E*/*A* < 1 or DT > 275 ms) or (*E*/*A* > 2.5 or DT < 110 ms)	• Diastolic dysfunction 19.4% HDF, 36.7% HD	• ↑ FS, EF in HDF v HD • ↑ Diastolic dysfunction HD v HDF
***Sgambat ***[61] Prospective longitudinal	• Transplant *n* = 40 • Mean 1mo post-Tx eGFR 94 ml/min/1.73 m^2^ • Pre tx echo and post tx • Healthy controls *n* = 24	• LVMI by mass /height^2.7^ • LVH = LVM/height^2.7^ ≥ 95th age based percentile • Systolic function by • FS by M-mode method, EF by 2D Simpson’s method • Systolic strain by STE oImpaired = > 95th percentile of controls	• LVH pre-Tx 37.1%, 1mo post 35.2%, 18mo 17.1%, 30mo 35.5% • ILS pre 76.7%, 1mo 47.1%, 18mo 40% • ICS pre21.2%, 1mo post 3%,18mo 3.1%	• ↑ LVMI, ↑ LS v controls • ↓ ILS 1 mo and 18 mo post KT v pre-KT • ↓ICS 1, 18, 30 mo post KT v pre-KT • ↑ LS associated with HD pre-transplant (no longer associated post-transplant) • ↑ S, LVH 1 mo post KT assoc with obesity • ↑LS associated with obesity/LDL interaction
***Yu ***[26] Retrospective longitudinal	• PD *n* = 35 • Mean duration PD at enrolment 2 ± 2y • Mean time between echos 1.3 ± 4.6y	• LVMI = mass /height^2.7^ / 95^th^ percentile for height age • LVH = LVMI ≥ 1.0 • LV geometry by LVH/RWT (defined by 0.43) • Diastolic function by • E,A; *E*′, *A*′; height indexed LAV	• LVH baseline 77%,follow-up 83% • Concentric LVH 51% at baseline, 71% at follow-up • Normal LV geometry 6% baseline, 3% follow-up	• LVMI change between scans correlated with diastolic function markers- LAV, A wave and Hb, urea
***Shroff ***[25] Non-randomised parallel-arm intervention	• HDF and HD *n* = 133 total • 80 incident, 53 prevalent • HD *n* = 78, HDF *n* = 55 • Inclusion age 5–20 y • Echo studies at 0 and 12 months whilst still on dialysis	• LVMI = mass/height • LVH by 95^th^ percentile	Not reported	• 12 mo ↑ in LVMI associated with • Intradialytic hypotension • ↑ interdialytic weight gain • ↑UF rate, ↑ MAP SD score • ↑ PTH,↑ BMI, ↓ HB • HD for incident patients
***Doan ***[56] Prospective observational	• HD *n* = 15 • Mean time on dialysis 2.0y • Echo studies pre-HD, mid-HD, post-HD	• Systolic function by EF, FS • Systolic strain by GLS (impaired defined by vendor RR) • Diastolic function by *E*/*A* and *E*/*E*′ • Diastolic strain by GLDs • LA Strain (LAS) and *E*/LAS	• ILS pre 53%, mid 100%, post 100%	• ↓ EF post v pre, mid v pre • ↓*E*/*A* post v pre, mid v pre • ↑ GLS post v pre, mid v pre • ↓ GLDs post v pre, post v mid, mid v pre • ↑ *E*/LAS reservoir post v pre, mid v pre • ↑ GLS post corr with higher mean BP drop

*KF*, kidney failure; *HD*, hemodialysis; *PD*, peritoneal dialysis; *CKD*, chronic kidney disease; *mo*, months; *y*, years; *LV*, left ventricle; *LVH*, left ventricular hypertrophy; *LVMI*, left ventricular mass index; *AD*, aortic dilation; *PWD*, pulse wave Doppler; *TDI*, tissue Doppler imaging; *RWT*, relative wall thickness; *abn*, abnormal; *ass*, associated; *corr*, correlated; *UF*, ultrafiltration; *HB*, hemoglobin; *PTH*, parathyroid hormone; *SBP*, systolic blood pressure; *MAP*, mean arterial pressure; *BP*, blood pressure; *DBP*, diastolic blood pressure; *BMI*, body mass index; *GFR*, glomerular filtration rate; *FS*, fractional shortening; *EF*, ejection fraction; *S′*, systolic myocardial velocity; *GLS*, global longitudinal strain; *LS*, longitudinal strain; *CS*, circumferential strain; *GLDs*, global longitudinal diastolic strain; *ILS*, impaired longitudinal strain; *ICS*, impaired circumferential strain; *HDF*, hemodiafiltration; *IVRT*, isovolumic relaxation time; *KT*, kidney transplant; *v*, versus; *HTN*, hypertension; *LAV*, left atrial volume; *LAS*, left atrial strain; *LDL*, low density lipoprotein

LVM/LVH is the most commonly studied echocardiogram parameter, with some studies also assessing LVM influence on function, demonstrating a correlation with worse diastolic [21, 59], and systolic [66] function. Hypertension has been consistently identified as a key risk factor for LVH in dialysis [8, 25, 58, 63, 64, 70] and transplant [70, 71] populations, with overweight/obese BMI also associated with LVH in dialysis [25] and transplant populations [61, 71]. LVM risk appears to increase from CKD to dialysis in children, although longitudinal studies are not available. In both peritoneal dialysis (PD) [65] and hemodialysis (HD) [8, 25] volume-related factors, anaemia [25, 58, 65] and hyperparathyroidism [21] are key risks. Where these are carefully managed, LVM and LV geometry can improve [57]. With transplantation, some regression in LVM can also be seen [72], although hypertension and metabolic risks can lead to persistent changes, or deterioration over time [71, 73]. A recent study has correlated LVM in children on dialysis with pulse wave velocity (PWV), a marker of vascular stiffness [74]. A direct causal relationship between PWV and LVM/LVH has not been established, and the correlation may simply reflect that hypertension is a common risk factor. CIMT is also associated with LVH in PD patients [65], again suggesting the presence of a common risk state. In this publication, inflammation was postulated as important given that both LVH and CIMT were significantly associated with CRP [65].

Global LV systolic function as measured by conventional echo-based LVEF/FS is mostly preserved in childhood chronic KF [35, 75]. During HD sessions, however, regional myocardial systolic dysfunction assessed by SF% (stunning) is a common finding [30]. In adults stunning has been demonstrated to progress to fixed regional systolic dysfunction and global reduction in LVEF by 12 months [31] and is associated with 12-month mortality [76]. Impaired GLS measured by STE has been demonstrated in childhood chronic KF in the absence of conventional echocardiography measures of systolic dysfunction [56]. In adult dialysis patients, GLS is associated with CV mortality independent of age and conventionally measured EF [43]. The HD population appears to be most at risk, especially if assessed during or immediately following a HD session [35, 47, 56, 77] and if there are more significant falls in systolic blood pressure or higher ultrafiltration volumes [56, 77]. Segmental differences in LV strain can also be seen at this time [47] including where measured by the asynchrony index (Table 1) [77]. This is a marker of LV mechanical dyssynchrony which is where there are regional differences in timing of ventricular contraction and relaxation. The asynchrony index is associated with ventricular arrythmias and sudden cardiac death in adult dialysis patients independent of LVEF [78]. A recent study using 2D STE post-hemodialysis has shown impaired LVEF derived by STE versus normal LVEF by conventional echocardiogram in children, providing further evidence of the sensitivity of STE [47]. Strain by STE or TDI has been shown in some pediatric transplant cohorts to be significantly worse than controls [35, 59], and has been associated with hypertension, metabolic syndrome, obesity, dyslipidaemia, lower EGFR, and past HD (versus PD or pre-emptive transplant) [61]. Longitudinal patient assessment has demonstrated that GLS can improve with transplantation [75], although it remains significantly worse versus controls [61]. Mitsnefes et al. have demonstrated evidence of LV hypercontractility in dialysis [8] and transplant patients [79]. In dialysis, the contractile reserve in exercise was reduced compared with controls, whereas in transplant patients the reserve was maintained. It was postulated that this could suggest an adaptive response perhaps mediated by sympathetic overactivity, and that over the long-term, this may be disadvantageous. To date, however, associated long-term outcomes have not been assessed to our knowledge. Kim et al. identified in the post-transplant cohort poorer LV performance, defined by the Tei index, and worse GLS by TDI, in the setting of increased FS and EF, and that this was associated with a longer time on dialysis prior to transplant [59]. Again, no longer-term follow-up data is available.

LV diastolic function can be impaired in both dialysis and transplantation [21, 41, 59, 60, 80]. Most studies evaluate this as a continuous parameter using the ratios *E*/*A*, *E*′/*A*′, or *E*/*E*′, although some define specific criteria for diastolic dysfunction [41, 60, 64, 66]. TDI measures of diastolic function may be more sensitive than PWD [41]. Duration of dialysis has been associated with markers of LV stiffness [80], and diastolic dysfunction (DD) may persist post-transplant [59, 60, 75]. DD has been associated with LVH/LVMI in HD [66] and transplant [67, 79]; however, this is not a consistent finding [41, 73], and could reflect how LVMI or DD was defined/measured, or other risk factors for DD in the studied population. Markers of volume overload [65], and hyperparathyroidism [21] have been associated with worse diastolic function. Risk post-transplant may be independent of hypertension [41, 73]. In adults, DD measured by *E*/*E*′ ratio has been associated with mortality [81]. In HD, left atrial strain (LAS) measures may be an even more sensitive measure of DD and cardiac events [82]. LAS has recently been demonstrated to worsen during HD in children [56].

Right heart changes are infrequently reported in childhood chronic KF, although these are still an important part of standard echocardiogram assessment. Right heart geometry has been reported to change in adolescents on HD, especially in the presence of an arterio-venous fistula [83], with increased right atrial and right ventricular (RV) free wall thickness and reduced RV volumes reported. This may be due to an increase in venous return.

Ascending aortic dilatation appears to be somewhat of a novel finding for children (versus adults) with chronic KF [68, 84, 85], and it is also seen in childhood CKD [86]. Poor nutrition may be an important risk factor [86]. Pre-emptive transplantation may be protective, and there appears to be an association with post-transplant hypertension [85]. We were unable to find publications with aortic measurements reported in adult chronic KF populations.

### Current guidelines for echocardiography use in kidney failure

Guidelines that incorporate routine echocardiography assessment of chronic kidney failure patients are listed in Table 3. These have mainly focused on pre-transplantation screening of adult patients to determine suitability, as cardiac mortality in the first few months post-transplant can be high [87]. Identification of ischaemic heart disease (IHD) via non-invasive tests including stress echocardiograms is the predominant focus, although this is controversial, especially for asymptomatic patients. The American Society of Transplantation guidelines incorporate LVEF into their risk stratification for progressing to non-invasive IHD screening in transplant candidates, with routine echocardiogram screening suggested for those who are identified at risk based on clinical screening, X-ray and ECG [88]. The NKK-KDOQI guidelines are the only guidelines that recommend more regular echocardiography in dialysis [89]. In addition, these are the only guidelines that reference children, in whom a resting echocardiogram is recommended in the first 3 months following dialysis initiation [89]. No guidelines address routine echocardiogram screening in transplant recipients.

**Table 3:** Established guidelines for echocardiogram use in kidney failure

Guideline	Recommendation
NKF KDOQI guidelines 2005 [89]	• Echocardiography should be performed in all patients at initiation of dialysis, once they have achieved dry weight (ideally within 1–3 months of dialysis initiation) and then at yearly intervals thereafter • Re-assessment is recommended with a change in clinical status (e.g. CHF symptoms, recurrent hypotension on dialysis, post cardiac events) or where considered for kidney transplant • Dry weight optimization should be achieved prior to testing, to enhance the interpretation of results • The interpretation of repeat echocardiographic evaluations should be done with consideration of the relationship between the echo exam and either the HD treatment or the presence or absence of PD fluid in the peritoneal cavity • Children commencing dialysis should be evaluated for the presence of cardiac disease (cardiomyopathy and valvular disease) using echocardiography once the patient has achieved dry weight (ideally within 3 months of the initiation of dialysis therapy)
KDIGO transplant candidate guideline 2020 [90]	• Resting echocardiogram screening is suggested for asymptomatic transplant candidates who have been on dialysis for at least 2 years or those who have risk factors for pulmonary hypertension • Non-invasive IHD screening including stress echocardiogram is suggested for asymptomatic candidates at high risk for coronary artery disease (CAD) (e.g. diabetes, previous CAD) or those with poor functional capacity
American Heart Association and the American College of Cardiology Foundation 2012 [91]	• It is reasonable to perform preoperative assessment of left ventricular function by echocardiography in potential kidney transplantation candidates • There is no evidence for or against surveillance by repeated left ventricular function tests after listing for kidney transplantation
American Society of Transplantation 2001 [88]	• LVH screening recommendations: (A) Patients should be evaluated for possible LVH with medical history, physical examination, electrocardiogram and chest X-ray (B) Patients with evidence of LVH should undergo an echocardiogram to confirm its presence and screen for possible underlying causes (A) Anaemia, hypertension, and IHD should be treated to reduce LVH and its associated complications • Screening for IHD recommendations: (A) Assess IHD risk factors: a prior history of IHD, men ≥ 45 or women ≥ 55 years, IHD in a first degree relative, current cigarette smoking, diabetes, hypertension, fasting total cholesterol > 200 mg/dl, high density lipoprotein cholesterol < 35 mg/dl and left ventricular hypertrophy (A) Risk factor modification should be aggressively pursued (B) Patients at high risk, e.g. kidney disease from diabetes, prior history of IHD, or ≥ 2 risk factors, should have a cardiac stress test (B) Patients with a positive cardiac stress test should undergo coronary angiography for possible revascularization prior to transplantation (B) Patients with critical coronary lesions should undergo revascularization prior to transplantation

### Should routine echocardiogram screening be recommended in pediatric kidney failure?

While prevalence of echocardiogram abnormalities is significant across childhood dialysis and transplant populations, current pediatric recommendations for echocardiogram screening in chronic KF are limited. A key screening test principle is that screening followed by an intervention should reduce clinical events. Currently, there are no trials assessing whether echocardiogram triggered interventions improve CV events. Physiologic principles, longitudinal studies, and multivariable analyses, including adult studies and those assessing echocardiogram outcomes, do, however, suggest that some interventions could be of benefit. For example, improved volume state and blood pressure in children is associated with better LVM in PD and HD [25, 63]. In adults, more frequent HD has been associated with reduced myocardial stunning [92], and in longitudinal studies, LVH regression with improved systolic function [93]. Shroff et al. demonstrated an increase in LVMI over 12 months for children on HD, with no significant change seen for patients on hemodiafiltration (HDF) [25], and Fadel identified an improvement in diastolic and systolic function for children converted to online-HDF with associated decrease in CRP [69]. In adults, some studies have shown improved survival with HDF [94]. Low hemoglobin has been associated with LVMI/LVH in childhood dialysis [25, 58], and in adults, correction of anaemia has been associated with regression of LVH [95]. Hemodialysate cooling was associated with less myocardial stunning in a randomised crossover trial in adults [96], and in a cross-sectional study of adults, stunning was rare in PD versus HD [97].

One could argue that many echocardiogram abnormalities are associated with volume overload or hypertension, and that a screening echocardiogram should not be required to identify these states. Regular echocardiogram screening to identify subclinical and potentially reversible end-organ damage could be of benefit, however, to prompt an earlier change in management and a more complete evaluation of patient CV risk and therapy options. This could be individualised with screening further extended or increased in frequency according to local echocardiogram access or baseline suspected risk. For example, HD patients could be screened during dialysis or immediately post-dialysis to identify stunning or strain. Given that stunning has been associated in adults with irreversible systolic dysfunction and mortality by 12 months [31], and there are indicators that strain is associated with myocardial fibrosis, any accessible intervention that has some level of evidence and is without harm should be considered. For example, change in modality to PD may be associated with less cumulative risk of stunning/strain, and more frequent HD or hemodialysate cooling may reduce stunning. Transplantation may also reduce these risks, with some evidence that impaired strain can be reversed [61]. Post-transplant, CV screening recommendations include metabolic and BP screening [98, 99], with ambulatory BP screening recommended due to the higher risk of masked hypertension in this population [99]. Of note, based on the American Association of Pediatrics (AAP) hypertension guidelines, any child with hypertension and kidney disease should have a yearly echocardiogram [99].

Minimum recommended standards for pediatric echocardiography have been published by the American Society of Echocardiography [39, 53]. These standards include all the important assessments from a chronic KF perspective, including measures of the left ventricle and aorta, an assessment of systolic function, and Doppler-based diastolic function. Given that longitudinal chronic KF studies identify evolving echocardiogram changes within 12 months [25, 61, 69, 70], a minimum of annual frequency of echocardiogram screening seems appropriate but can be adjusted according to findings and risk factors.

## Conclusions

Given future lifelong exaggerated CV risks in childhood chronic KF, and the complexities of these risk states, it is our opinion that regular assessment of CV risk factors and subclinical CV health is warranted during dialysis, pre- and post-transplant. Annual echocardiography, even with minimum standard assessment, is well-placed to help screen overall cardiac health. Further extended screening using newer modalities such as STE could be considered dependent on baseline suspected or known CV risk status. Echocardiography is a well-established technique which does not involve risk or significant time commitment for patients, and also provides important functional data. Further long-term research and interventional studies would help further clarify the specific utility of echocardiogram screening in the childhood chronic KF population.

## References

[1] McDonald SP, Craig JC (2004). Long-term survival of children with end-stage renal disease. N Engl J Med.

[2] Parekh RS, Carroll CE, Wolfe RA, Port FK (2002). Cardiovascular mortality in children and young adults with end-stage kidney disease. J Pediatr.

[3] Meier-Kriesche HU, Schold JD, Srinivas TR, Reed A, Kaplan B (2004). Kidney transplantation halts cardiovascular disease progression in patients with end-stage renal disease. Am J Transplant.

[4] Genovesi S, Boriani G, Covic A, Vernooij RWM, Combe C, Burlacu A (2021). Sudden cardiac death in dialysis patients: different causes and management strategies. Nephrol Dial Transplant.

[5] Amann K, Rychlík I, Miltenberger-Milteny G, Ritz E (1998). Left ventricular hypertrophy in renal failure. Kidney Int Suppl.

[6] London GM (2000). Alterations of arterial function in end-stage renal disease. Nephron.

[7] Rong S, Qiu X, Jin X, Shang M, Huang Y, Tang Z (2018). Risk factors for heart valve calcification in chronic kidney disease. Medicine (Baltimore).

[8] Mitsnefes MM, Kimball TR, Witt SA, Glascock BJ, Khoury PR, Daniels SR (2003). Left ventricular mass and systolic performance in pediatric patients with chronic renal failure. Circulation.

[9] Mitsnefes MM (2021). Cardiovascular disease risk factors in chronic kidney disease in children. Semin Nephrol.

[10] Mitsnefes M, Stablein D (2005). Hypertension in pediatric patients on long-term dialysis: a report of the North American Pediatric Renal Transplant Cooperative Study (NAPRTCS). Am J Kidney Dis.

[11] Sinha MD, Kerecuk L, Gilg J, Reid CJ (2012). Systemic arterial hypertension in children following renal transplantation: prevalence and risk factors. Nephrol Dial Transplant.

[12] Silverstein DM (2004). Risk factors for cardiovascular disease in pediatric renal transplant recipients. Pediatr Transplant.

[13] Shroff R, Speer T, Colin S, Charakida M, Zewinger S, Staels B (2014). HDL in children with CKD promotes endothelial dysfunction and an abnormal vascular phenotype. J Am Soc Nephrol.

[14] Goodman WG, Goldin J, Kuizon BD, Yoon C, Gales B, Sider D (2000). Coronary-artery calcification in young adults with end-stage renal disease who are undergoing dialysis. N Engl J Med.

[15] Srivaths PR, Silverstein DM, Leung J, Krishnamurthy R, Goldstein SL (2010). Malnutrition-inflammation-coronary calcification in pediatric patients receiving chronic hemodialysis. Hemodial Int.

[16] Paik JK, Kim M, Kwak JH, Lee EK, Lee SH, Lee JH (2013). Increased arterial stiffness in subjects with impaired fasting glucose. J Diabetes Complications.

[17] Cobo G, Lindholm B, Stenvinkel P (2018). Chronic inflammation in end-stage renal disease and dialysis. Nephrol Dial Transplant.

[18] Oh J, Wunsch R, Turzer M, Bahner M, Raggi P, Querfeld U (2002). Advanced coronary and carotid arteriopathy in young adults with childhood-onset chronic renal failure. Circulation.

[19] Panichi V, Rizza GM, Paoletti S, Bigazzi R, Aloisi M, Barsotti G (2008). Chronic inflammation and mortality in haemodialysis: effect of different renal replacement therapies. Results from the RISCAVID study. Nephrol Dial Transplant.

[20] Dekker MJE, van der Sande FM, van den Berghe F, Leunissen KML, Kooman JP (2018). Fluid overload and inflammation axis. Blood Purif.

[21] Mitsnefes MM, Kimball TR, Kartal J, Witt SA, Glascock BJ, Khoury PR (2005). Cardiac and vascular adaptation in pediatric patients with chronic kidney disease: role of calcium-phosphorus metabolism. J Am Soc Nephrol.

[22] Stevens KK, McQuarrie EP, Sands W, Hillyard DZ, Patel RK, Mark PB (2011). Fibroblast growth factor 23 predicts left ventricular mass and induces cell adhesion molecule formation. Int J Nephrol.

[23] Litwin M, Wühl E, Jourdan C, Trelewicz J, Niemirska A, Fahr K (2005). Altered morphologic properties of large arteries in children with chronic renal failure and after renal transplantation. J Am Soc Nephrol.

[24] Civilibal M, Caliskan S, Adaletli I, Oflaz H, Sever L, Candan C (2006). Coronary artery calcifications in children with end-stage renal disease. Pediatr Nephrol.

[25] Shroff R, Smith C, Ranchin B, Bayazit AK, Stefanidis CJ, Askiti V (2019). Effects of hemodiafiltration versus conventional hemodialysis in children with ESKD: the HDF, heart and height study. J Am Soc Nephrol.

[26] Yu JJ, Jun HO, Shin EJ, Baek JS, Lee JH, Kim YH (2018). Factors associated with reduction of left ventricular mass in children on peritoneal dialysis. Nephrology (Carlton).

[27] Radhakrishnan A, Pickup LC, Price AM, Law JP, McGee KC, Fabritz L (2021). Coronary microvascular dysfunction is associated with degree of anaemia in end-stage renal disease. BMC Cardiovasc Disord.

[28] Amaral S, Sayed BA, Kutner N, Patzer RE (2016). Preemptive kidney transplantation is associated with survival benefits among pediatric patients with end-stage renal disease. Kidney Int.

[29] Chou JA, Streja E, Nguyen DV, Rhee CM, Obi Y, Inrig JK (2018). Intradialytic hypotension, blood pressure changes and mortality risk in incident hemodialysis patients. Nephrol Dial Transplant.

[30] Hothi DK, Rees L, Marek J, Burton J, McIntyre CW (2009). Pediatric myocardial stunning underscores the cardiac toxicity of conventional hemodialysis treatments. Clin J Am Soc Nephrol.

[31] Burton JO, Jefferies HJ, Selby NM, McIntyre CW (2009). Hemodialysis-induced repetitive myocardial injury results in global and segmental reduction in systolic cardiac function. Clin J Am Soc Nephrol.

[32] Georgianos PI, Agarwal R (2015). Relative importance of aortic stiffness and volume as predictors of treatment-induced improvement in left ventricular mass index in dialysis. PLoS ONE.

[33] Gaasch WH, Zile MR (2011). Left ventricular structural remodeling in health and disease: with special emphasis on volume, mass, and geometry. J Am Coll Cardiol.

[34] Shroff RC, Donald AE, Hiorns MP, Watson A, Feather S, Milford D (2007). Mineral metabolism and vascular damage in children on dialysis. J Am Soc Nephrol.

[35] van Huis M, Schoenmaker NJ, Groothoff JW, van der Lee JH, van Dyk M, Gewillig M (2016). Impaired longitudinal deformation measured by speckle-tracking echocardiography in children with end-stage renal disease. Pediatr Nephrol.

[36] Foster BJ, Khoury PR, Kimball TR, Mackie AS, Mitsnefes M (2016). New reference centiles for left ventricular mass relative to lean body mass in children. J Am Soc Echocardiogr.

[37] Foster BJ, Mackie AS, Mitsnefes M, Ali H, Mamber S, Colan SD (2008). A novel method of expressing left ventricular mass relative to body size in children. Circulation.

[38] Tei C (1995). New non-invasive index for combined systolic and diastolic ventricular function. J Cardiol.

[39] Lopez L, Colan SD, Frommelt PC, Ensing GJ, Kendall K, Younoszai AK (2010). Recommendations for quantification methods during the performance of a pediatric echocardiogram: a report from the Pediatric Measurements Writing Group of the American Society of Echocardiography Pediatric and Congenital Heart Disease Council. J Am Soc Echocardiogr.

[40] Sohn DW, Chai IH, Lee DJ, Kim HC, Kim HS, Oh BH (1997). Assessment of mitral annulus velocity by Doppler tissue imaging in the evaluation of left ventricular diastolic function. J Am Coll Cardiol.

[41] Schoenmaker NJ, Kuipers IM, van der Lee JH, Tromp WF, van Dyck M, Gewillig M (2014). Diastolic dysfunction measured by tissue Doppler imaging in children with end-stage renal disease: a report of the RICH-Q study. Cardiol Young.

[42] Eidem BW, McMahon CJ, Cohen RR, Wu J, Finkelshteyn I, Kovalchin JP (2004). Impact of cardiac growth on Doppler tissue imaging velocities: a study in healthy children. J Am Soc Echocardiogr.

[43] Kramann R, Erpenbeck J, Schneider RK, Röhl AB, Hein M, Brandenburg VM (2014). Speckle tracking echocardiography detects uremic cardiomyopathy early and predicts cardiovascular mortality in ESRD. J Am Soc Nephrol.

[44] Krämer J, Niemann M, Liu D, Hu K, Machann W, Beer M (2013). Two-dimensional speckle tracking as a non-invasive tool for identification of myocardial fibrosis in Fabry disease. Eur Heart J.

[45] Dubin RF, Guajardo I, Ayer A, Mills C, Donovan C, Beussink L (2016). Associations of macro- and microvascular endothelial dysfunction with subclinical ventricular dysfunction in end-stage renal disease. Hypertension.

[46] Terhuerne J, van Diepen M, Kramann R, Erpenbeck J, Dekker F, Marx N (2021). Speckle-tracking echocardiography in comparison with ejection fraction for prediction of cardiovascular mortality in patients with end-stage renal disease. Clin Kidney J.

[47] Rakha S, Hafez M, Bakr A, Hamdy N (2020). Changes of cardiac functions after hemodialysis session in pediatric patients with end-stage renal disease: conventional echocardiography and two-dimensional speckle tracking study. Pediatr Nephrol.

[48] Muraru D, Niero A, Rodriguez-Zanella H, Cherata D, Badano L (2018). Three-dimensional speckle-tracking echocardiography: benefits and limitations of integrating myocardial mechanics with three-dimensional imaging. Cardiovasc Diagn Ther.

[49] Kutty S, Biko DM, Goldberg AB, Quartermain MD, Feinstein SB (2021). Contrast-enhanced ultrasound in pediatric echocardiography. Pediatr Radiol.

[50] Mese T, Guven B, Yilmazer MM, Serdaroglu E, Tavli V, Haydar A (2010). Contractility reserve in children undergoing dialysis by dobutamine stress echocardiography. Pediatr Cardiol.

[51] Russell K, Eriksen M, Aaberge L, Wilhelmsen N, Skulstad H, Remme EW (2012). A novel clinical method for quantification of regional left ventricular pressure-strain loop area: a non-invasive index of myocardial work. Eur Heart J.

[52] Chen KW, Hsieh WT, Huang CY, Huang CC, Liang HY, Wang GJ (2021). Estimated left ventricular pressure-myocardial strain loop as an index of cardiac work predicts all-cause mortality in patients receiving regular hemodialysis. J Diabetes Complications.

[53] Lai WW, Geva T, Shirali GS, Frommelt PC, Humes RA, Brook MM (2006). Guidelines and standards for performance of a pediatric echocardiogram: a report from the Task Force of the Pediatric Council of the American Society of Echocardiography. J Am Soc Echocardiogr.

[54] Ie EH, Vletter WB, ten Cate FJ, Nette RW, Weimar W, Roelandt JR (2003). Preload dependence of new Doppler techniques limits their utility for left ventricular diastolic function assessment in hemodialysis patients. J Am Soc Nephrol.

[55] Hirayama S, Ando Y, Sudo Y, Asano Y (2002). Improvement of cardiac function by dry weight optimization based on interdialysis inferior vena caval diameter. ASAIO J.

[56] Doan TT, Srivaths P, Liu A, Kevin Wilkes J, Idrovo A, Akcan-Arikan A (2021). Left ventricular strain and left atrial strain are impaired during hemodialysis in children. Int J Cardiovasc Imaging.

[57] Melhem N, Savis A, Wheatley A, Copeman H, Willmott K, Reid CJD (2019). Improved blood pressure and left ventricular remodelling in children on chronic intermittent haemodialysis: a longitudinal study. Pediatr Nephrol.

[58] Civilibal M, Caliskan S, Oflaz H, Sever L, Candan C, Canpolat N (2007). Traditional and “new” cardiovascular risk markers and factors in pediatric dialysis patients. Pediatr Nephrol.

[59] Kim GB, Kwon BS, Kang HG, Ha JW, Ha IS, Noh CI (2009). Cardiac dysfunction after renal transplantation; incomplete resolution in pediatric population. Transplantation.

[60] Lindblad YT, Axelsson J, Balzano R, Vavilis G, Chromek M, Celsi G (2013). Left ventricular diastolic dysfunction by tissue Doppler echocardiography in pediatric chronic kidney disease. Pediatr Nephrol.

[61] Sgambat K, Clauss S, Lei KY, Song J, Rahaman SO, Lasota M (2018). Effects of obesity and metabolic syndrome on cardiovascular outcomes in pediatric kidney transplant recipients: a longitudinal study. Pediatr Nephrol.

[62] Mitsnefes MM, Daniels SR, Schwartz SM, Meyer RA, Khoury P, Strife CF (2000). Severe left ventricular hypertrophy in pediatric dialysis: prevalence and predictors. Pediatr Nephrol.

[63] Mitsnefes MM, Daniels SR, Schwartz SM, Khoury P, Strife CF (2001). Changes in left ventricular mass in children and adolescents during chronic dialysis. Pediatr Nephrol.

[64] Gruppen MP, Groothoff JW, Prins M, van der Wouw P, Offringa M, Bos WJ (2003). Cardiac disease in young adult patients with end-stage renal disease since childhood: a Dutch cohort study. Kidney Int.

[65] Bakkaloglu SA, Saygili A, Sever L, Noyan A, Akman S, Ekim M (2009). Assessment of cardiovascular risk in paediatric peritoneal dialysis patients: a Turkish Pediatric Peritoneal Dialysis Study Group (TUPEPD) report. Nephrol Dial Transplant.

[66] Shamszad P, Slesnick TC, Smith EO, Taylor MD, Feig DI (2012). Association between left ventricular mass index and cardiac function in pediatric dialysis patients. Pediatr Nephrol.

[67] Hirth A, Edwards NC, Greve G, Tangeraas T, Gerdts E, Lenes K (2012). Left ventricular function in children and adults after renal transplantation in childhood. Pediatr Nephrol.

[68] Kaddourah A, Uthup S, Madueme P, O'Rourke M, Hooper DK, Taylor MD (2015). Prevalence and predictors of aortic dilation as a novel cardiovascular complication in children with end-stage renal disease. Clin Nephrol.

[69] Fadel FI, Makar SH, Zekri H, Ahmed DH, Aon AH (2015). The effect of on-line hemodiafiltration on improving the cardiovascular function parameters in children on regular dialysis. Saudi J Kidney Dis Transpl.

[70] Mitsnefes MM, Schwartz SM, Daniels SR, Kimball TR, Khoury P, Strife CF (2001). Changes in left ventricular mass index in children and adolescents after renal transplantation. Pediatr Transplant.

[71] Hamdani G, Nehus EJ, Hanevold CD, Sebestyen Van Sickle J, Woroniecki R, Wenderfer SE (2017). Ambulatory blood pressure, left ventricular hypertrophy, and allograft function in children and young adults after kidney transplantation. Transplantation.

[72] Becker-Cohen R, Nir A, Ben-Shalom E, Rinat C, Feinstein S, Farber B (2008). Improved left ventricular mass index in children after renal transplantation. Pediatr Nephrol.

[73] Basiratnia M, Esteghamati M, Ajami GH, Amoozgar H, Cheriki C, Soltani M (2011). Blood pressure profile in renal transplant recipients and its relation to diastolic function: tissue Doppler echocardiographic study. Pediatr Nephrol.

[74] Filip C, Cirstoveanu C, Bizubac M, Berghea EC, Căpitănescu A, Bălgrădean M (2021). Pulse wave velocity as a marker of vascular dysfunction and its correlation with cardiac disease in children with end-stage renal disease (ESRD). Diagnostics (Basel).

[75] Rumman RK, Ramroop R, Chanchlani R, Ghany M, Hebert D, Harvey EA (2017). Longitudinal assessment of myocardial function in childhood chronic kidney disease, during dialysis, and following kidney transplantation. Pediatr Nephrol.

[76] Burton JO, Jefferies HJ, Selby NM, McIntyre CW (2009). Hemodialysis-induced cardiac injury: determinants and associated outcomes. Clin J Am Soc Nephrol.

[77] Hothi DK, Rees L, McIntyre CW, Marek J (2013). Hemodialysis-induced acute myocardial dyssynchronous impairment in children. Nephron Clin Pract.

[78] Hensen LCR, Goossens K, Podlesnikar T, Rotmans JI, Jukema JW, Delgado V (2018). Left ventricular mechanical dispersion and global longitudinal strain and ventricular arrhythmias in predialysis and dialysis patients. J Am Soc Echocardiogr.

[79] Mitsnefes MM, Kimball TR, Border WL, Witt SA, Glascock BJ, Khoury PR (2004). Abnormal cardiac function in children after renal transplantation. Am J Kidney Dis.

[80] Choi AW, Fong NC, Li VW, Ho TW, Chan EY, Ma AL (2020). Left ventricular stiffness in paediatric patients with end-stage kidney disease. Pediatr Nephrol.

[81] Farshid A, Pathak R, Shadbolt B, Arnolda L, Talaulikar G (2013). Diastolic function is a strong predictor of mortality in patients with chronic kidney disease. BMC Nephrol.

[82] Tsai WC, Lee WH, Wu PY, Huang JC, Chen YC, Chen SC (2019). Ratio of transmitral E wave velocity to left atrial strain as a useful predictor of total and cardiovascular mortality in hemodialysis patients. J Clin Med.

[83] Çakıcı EK, Çakıcı M, Gümüş F, Tan Kürklü TS, Yazılıtaş F, Örün UA (2020). Effects of hemodialysis access type on right heart geometry in adolescents. J Vasc Access.

[84] Quennelle S, Ovaert C, Cailliez M, Garaix F, Tsimaratos M, El Louali F (2021). Dilatation of the aorta in children with advanced chronic kidney disease. Pediatr Nephrol.

[85] Surak A, Filler G, Sharma AP, Torres Canchala LA, Grattan M (2020). Lower prevalence of aortic dilatation among preemptive pediatric renal transplant recipients - a cross-sectional cohort study. Pediatr Transplant.

[86] Madueme PC, Ng DK, Guju L, Longshore L, Moore V, Jefferies L (2020). Aortic dilatation in children with mild to moderate chronic kidney disease. Pediatr Nephrol.

[87] Wyld MLR, De La Mata NL, Masson P, O'Lone E, Kelly PJ, Webster AC (2021). Cardiac mortality in kidney transplant patients: a population-based cohort study 1988–2013 in Australia and New Zealand. Transplantation.

[88] Kasiske BL, Cangro CB, Hariharan S, Hricik DE, Kerman RH, Roth D (2001). The evaluation of renal transplantation candidates: clinical practice guidelines. Am J Transplant.

[89] K/DOQI Working Group (2005). K/DOQI clinical practice guidelines for cardiovascular disease in dialysis patients. Am J Kidney Dis.

[90] Chadban SJ, Ahn C, Axelrod DA, Foster BJ, Kasiske BL, Kher V (2020). KDIGO Clinical practice guideline on the evaluation and management of candidates for kidney transplantation. Transplantation.

[91] Lentine KL, Costa SP, Weir MR, Robb JF, Fleisher LA, Kasiske BL (2012). Cardiac disease evaluation and management among kidney and liver transplantation candidates: a scientific statement from the American Heart Association and the American College of Cardiology Foundation: endorsed by the American Society of Transplant Surgeons, American Society of Transplantation, and National Kidney Foundation. Circulation.

[92] Jefferies HJ, Virk B, Schiller B, Moran J, McIntyre CW (2011). Frequent hemodialysis schedules are associated with reduced levels of dialysis-induced cardiac injury (myocardial stunning). Clin J Am Soc Nephrol.

[93] Susantitaphong P, Koulouridis I, Balk EM, Madias NE, Jaber BL (2012). Effect of frequent or extended hemodialysis on cardiovascular parameters: a meta-analysis. Am J Kidney Dis.

[94] Maduell F, Moreso F, Pons M, Ramos R, Mora-Macià J, Carreras J (2013). High-efficiency postdilution online hemodiafiltration reduces all-cause mortality in hemodialysis patients. J Am Soc Nephrol.

[95] Cannella G, La Canna G, Sandrini M, Gaggiotti M, Nordio G, Movilli E (1991). Reversal of left ventricular hypertrophy following recombinant human erythropoietin treatment of anaemic dialysed uraemic patients. Nephrol Dial Transplant.

[96] Selby NM, Burton JO, Chesterton LJ, McIntyre CW (2006). Dialysis-induced regional left ventricular dysfunction is ameliorated by cooling the dialysate. Clin J Am Soc Nephrol.

[97] Selby NM, McIntyre CW (2011). Peritoneal dialysis is not associated with myocardial stunning. Perit Dial Int.

[98] Chapman JR (2010). The KDIGO clinical practice guidelines for the care of kidney transplant recipients. Transplantation.

[99] Flynn JT, Kaelber DC, Baker-Smith CM, Blowey D, Carroll AE, Daniels SR, de Ferranti SD, Dionne JM, Falkner B, Flinn SK, Gidding SS, Goodwin C, Leu MG, Powers ME, Rea C, Samuels J, Simasek M, Thaker VV, Urbina EM (2017) Clinical practice guideline for screening and management of high blood pressure in children and adolescents. Pediatrics 140. 10.1542/peds.2017-1904 10.1542/peds.2017-1904 28827377

